# Interactions between Mannosylerythritol Lipid-A and Heat-Induced Soy Glycinin Aggregates: Physical and Chemical Characteristics, Functional Properties, and Structural Effects

**DOI:** 10.3390/molecules27217393

**Published:** 2022-10-31

**Authors:** Siyu Liu, Tianyu Wei, Hongyun Lu, Xiayu Liu, Ying Shi, Qihe Chen

**Affiliations:** Department of Food Science and Nutrition, Zhejiang University, Hangzhou 310058, China

**Keywords:** glycinin aggregation, mannosylerythritol lipid-A (MEL-A), interfacial performance

## Abstract

Protein-surfactant interactions have a significant influence on food functionality, which has attracted increasing attention. Herein, the effect of glycolipid mannosylerythritol lipid-A (MEL-A) on the heat-induced soy glycinin (11S) aggregates was investigated by measuring the structure, binding properties, interfacial behaviors, and emulsification characteristics of the aggregates. The results showed that MEL-A led to a decrease in the surface tension, viscoelasticity, and foaming ability of the 11S aggregates. In addition, MEL-A with a concentration above critical micelle concentration (CMC) reduced the random aggregation of 11S protein after heat treatment, thus facilitating the formation of self-assembling core-shell particles composed of a core of 11S aggregates covered by MEL-A shells. Infrared spectroscopy, circular dichroism spectroscopy, fluorescence spectroscopy, and isothermal titration calorimetry also confirmed that the interaction forces between MEL-A and 11S were driven by hydrophobic interactions between the exposed hydrophobic groups of the protein and the fatty acid chains or acetyl groups of MEL-A, as well as the hydrogen bonding between mannosyl-D-erythritol groups of MEL-A and amino acids of 11S. The findings of this study indicated that such molecular interactions are responsible for the change in surface behavior and the enhancement of foaming stability and emulsifying property of 11S aggregates upon heat treatment.

## 1. Introduction

11S is the main component of soy protein, accounting for 50–90% of the mass fraction of soy protein. 11S protein is a hexamer composed of six subunits whose molecular mass is 300–380 kDa [1]. Each subunit consists of an acidic subunit A (acidic pI) and a basic subunit B (basic pI) connected by a disulfide bond [2,3]. The amino acid sequence analysis showed that the acidic subunit contains more hydrophilic amino acids, while the basic subunit contains more hydrophobic amino acids [4]. Based on the typical amphiphilic structure and its advantages for health [5,6], soybean protein can be used as an effective foam or emulsion stabilizer in the food industry. However, numerous studies have shown that the foaming and emulsifying ability of soy protein is much weaker than casein and whey protein due to its low surface hydrophobicity, high molecular flexibility, and large molecular size [7,8]. These factors inhibit the rapid adsorption of soy protein macromolecules at the air-water and the oil-water interfaces, making them less capable of foaming and emulsifying [9]. Currently, food scientists modify proteins by using their own structures and grafting them onto polysaccharides (electrostatic complexes and covalent grafts) and surfactants with low molecular weight (LMW) to enhance the foaming or emulsifying properties of soy protein [10,11,12,13]. Therefore, a better understanding of the aggregation process of 11S is necessary and feasible to control the formation of food structures and improve the properties of soy products.

Surfactants are a class of amphiphilic molecules, usually consisting of an oleophilic group or its substituents and a hydrophilic group. Surfactants exhibit good surface activity and interfacial performances on gas, solid, and liquid surfaces [14]. At a certain concentration, surfactants aggregate and form ordered subsets, monomolecular layers, micelles, vesicles, and sublayers on the surfaces [15]. They are widely used in the food industry due to their activating effects, such as dispersibility and emulsification. Compared to macromolecular proteins, LMW surfactants can migrate from the bulk phase solution to the interface more rapidly and show stronger surface activity, thus reducing the interfacial tension more effectively and facilitating foam or emulsion formation due to the Gibbs–Marangoni mechanism [16]. However, the system of small molecule surfactants is unstable and easy to polymerize. Therefore, small-molecule surfactants are usually used in combination with large-molecule proteins in the processing of food systems such as foams and emulsions, and their interfacial adsorption characteristics are reasonably utilized to prepare food systems with good forming ability and long-term stability [17,18]. Most surfactants are synthesized by chemical methods from petroleum, causing the consumption of resources and some serious environmental problems [19,20]. Therefore, it is imperative to develop green and clean preparation methods as well as products of surfactants.

Biosurfactants are compounds produced by microorganisms with amphiphilic and good surface activity [21]. Biosurfactants have multiple properties better than chemical surfactants, such as being biodegradable and environmentally compatible, safe, non-toxic or low-toxic [22,23], and mild production conditions [24]. Among them, mannosylerythritol lipids (MELs) are a class of glycolipids biosurfactants with polar 4-O-β-D-mannopyranosyl-D-erythritol and nonpolar fatty acid acyl chains. MELs can self-assemble into lyotropic liquid crystal phases, large unilamellar vesicles, and sponge structures depending on their concentration [25]. MELs have not only good emulsification, degradability, and low critical micelle concentration but also many special physiological activities. MELs inhibit the growth of Gram-positive bacteria, promoting cell injury and rupture [26] and favor the increase in the transfection efficiency of liposomes. Such modifications could change their interfacial properties and, thus, reduce the surface tension, enhance the solubility, lower critical micelle concentrations, and form unique self-assembly structures [27]. There are some recent studies on the interaction of 11S and soyasaponin mixtures [28].

With the increasing application of 11S in the food industry, it is of great importance to understand the binding properties of MEL-A with 11S and its effect on the formation of heat-induced 11S aggregates. Thus, the aim of this study was to evaluate the influences of MEL-A on the process of 11S aggregate formation under a heating treatment environment and the interaction between MEL-A and 11S. Furthermore, the interfacial behaviors, the foaming and emulsifying properties of 11S-MEL-A mixtures were investigated in this paper.

## 2. Results

### 2.1. Dynamic Surface Tension of 11S-MEL-A Mixtures upon Heating Treatment

**Figure 1:**
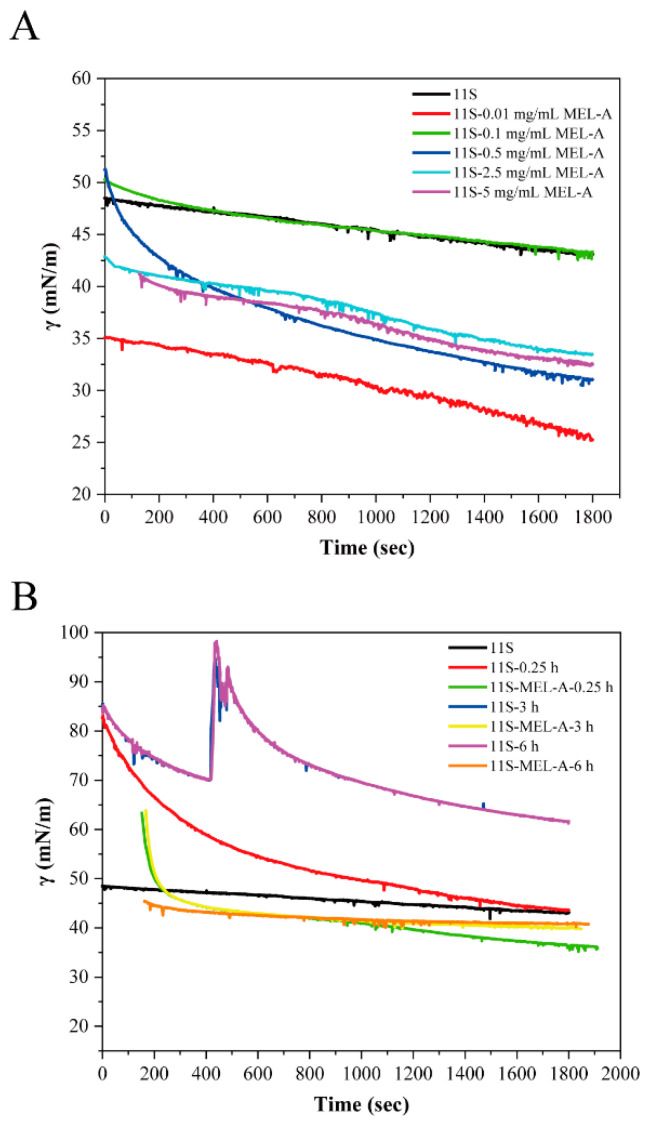
(**A**) The surface tension changes of heat-treated 11S and 11S-MEL-A mixtures adsorption at the air–water face with time. (**B**) Surface tension versus the concentrations of MEL-A during adsorption.

In this work, we conducted the effect of MEL-A on the surface tension improvement of 11S protein after heating treatment. The interfacial properties of surfactant and protein are closely related to the formation and stabilization of foams or emulsions. Hence, we needed to explore and analyze the interfacial properties of 11S-MEL-A mixtures and heated 11S. The dynamic surface tension (γ) of 11S with different concentrations of MEL-A (Figure 1A) showed a similar downward trend, which was attributed to the surface-active substances of MEL-A and 11S adsorption at the air-water interface. With the extension of adsorption time, the surface layer composed of 11S and Mel-A gradually saturated and stabilized, and the downward trend tended to be gentle. At the concentration of 0.1 mg/mL, the dynamic surface tension curves of 11S-MEL-A and pure 11S were the same. When the concentration of MEL-A increased to more than 2.5 mg/mL, the surface tension of the mixture decreased slightly. After heat treatment, the surface tension of 11S decreased sharply, indicating that heat treatment affected its surface activity to a large extent (Figure 1B). However, the surface tension of the 11S-MEL-A mixture was weaker than that of pure 11S, which might be related to the formation of the mixture layer of MEL-A and 11S. With the increase of the MEL-A/11S ratio, MEL-A may play a dominant role at the interface and gradually replace the protein. The adsorption behavior of surfactants generally refers to the formation and stability of the emulsion system. The co-adsorption of surfactant on the protein layer at the interface causes the change in the properties of the adsorbed layer and the complex. The long-term stability of the system can be induced by regulating the mixture of protein–saponin, which has potential implications for the application of natural food additives in emulsion design [28].

### 2.2. Foaming and Emulsifying Properties of 11S-MEL-A Mixtures upon Heating Treatment

**Figure 2:**
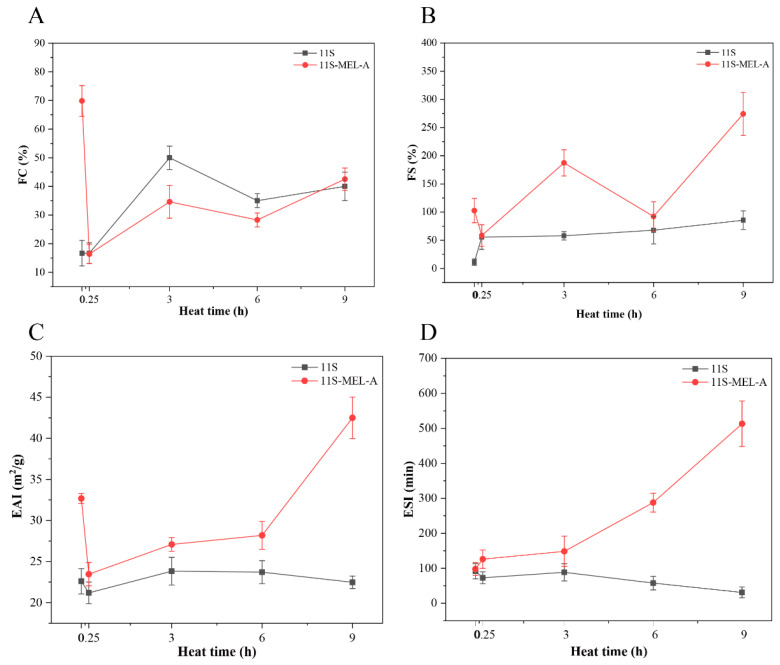
FC (**A**) and FS (**B**) of the 11S and 11S-MEL-A mixtures depend on the heat treatment time. EAI (**C**) and ESI (**D**) of the 11S and 11S-MEL-A mixtures as a function of the heat treatment time.

In most food and beverage systems, proteins play roles in stabilizing foams and emulsions. Some reports have shown that heating 11S protein can improve its emulsifying and foaming properties [29,30]. It is vital to study the effect of 11S aggregates on foaming stability and emulsifying stability. 11S solution showed poor foaming capacity (FC) with a percentage of 16% (Figure 2A). The large spherical conformation of 11S may influence the formation of the foam, which is related to the adsorption rate of surfactant at the air-water interface and the ability to reduce surface tension. Since the rate of protein adsorption depends on the molecular weight and structure of the protein, disordered, smaller, more elastic proteins are favorable for foam formation, and the large spherical conformation of 11S is not conducive to the formation of foam. Upon heat-treated samples, 11S showed an increased FC, indicating that the heat-denatured protein had a more flexible structure and stronger foaming ability. Compared with 11s and unheated 11S-MEL-A, the mixture of 11S-MEL-A showed lower FC after 3 h and 6 h heat treatment, which can be inferred that the foaming ability of the new 11S-MEL-A complex is low. However, the 11S-MEL-A mixture exhibited better foaming stability (FS) without and after heating (Figure 2B). According to other reports, excessive molecular aggregation is detrimental to the foaming and stability of 11S, while the presence of MEL-A reduces the formation of 11S aggregates, promotes structural changes and makes the mixture more stable [16,28]. This result is consistent with the enhancement of the foaming properties of other surfactant protein systems. Through the Gibbs–Marangoni mechanism, the reason could be considered the reaction model in which the surfactant stabilizes the interface and the protein forms a thick interface layer to protect the droplets from coalescence [11]. In addition, the mechanism of action was closely related to the proportion of surfactants and emulsifiers. The addition of MEL-A promoted the formation of a flowing adsorption layer of protein, which has a good response to the external deformation, thus preventing the rupture of the interface membrane and finally maintaining the stability of the foam. As shown in Figure 2C,D, the emulsifying activity of 11S changed little with the increase in heating time. However, the heating time of 0.25–6.0 h destroyed the stability of the emulsion system and decreased the stability of the emulsion phase. The results demonstrated that the denatured proteins heated for a long time are more likely to form flocculation or aggregation between the emulsion droplets. In the short heating time (0–6 h), the emulsifying activity and emulsifying stability of the 11S-MEL-A mixture were greatly higher than that of 11S. These data indicated that the increase in emulsifying ability was beneficial to the formation of small droplets with high stability. Combined with the findings of the infrared spectrum and other experimental data, 11S is denatured under the thermal condition due to more exposure to the stealthy hydrophobic group and thiol group. Due to the presence of MEL-A, the new complexes have flexible hydrophilic sugar groups and hydrophobic chains. Because the emulsifying properties of proteins are closely related to their structures, the more elastic 11S-MEL-A complex is beneficial to the emulsifying activity and stability of proteins. The interaction of MEL-A, which is composed of fatty acid chains, may improve the hydrophobicity of the emulsion and thus increase the emulsifying activity index (EAI) and emulsifying stability index (ESI) values of the emulsion.

### 2.3. The Effects of MEL-A on the Structure of 11S upon Heating

**Figure 3:**
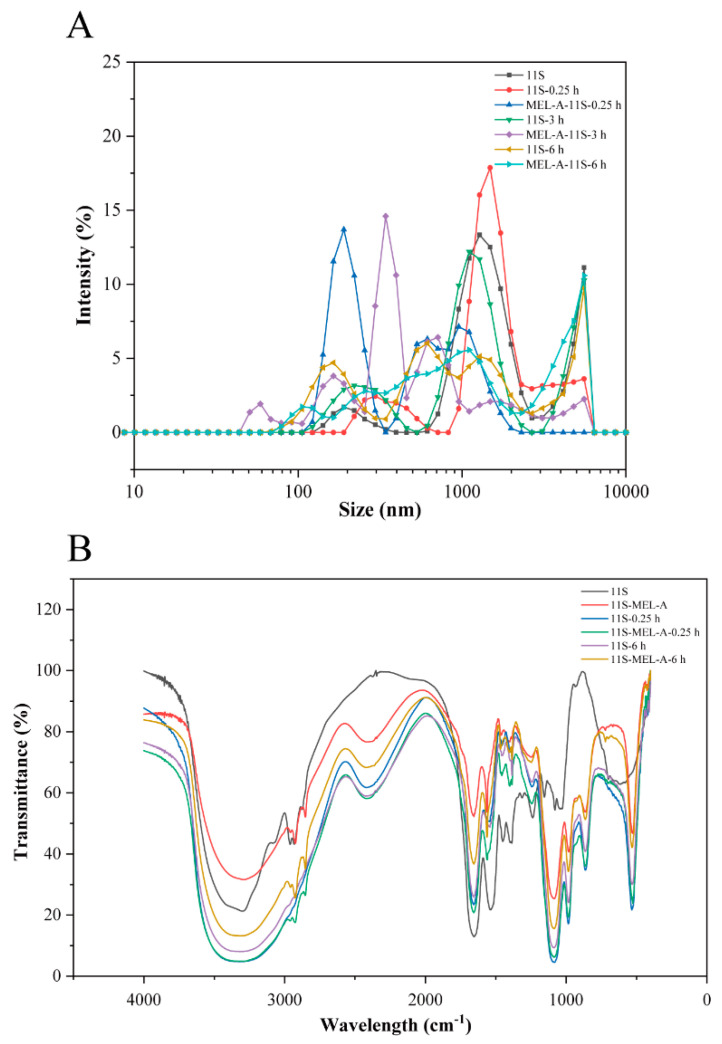
Effect of MEL-A on molecular size and chemical structure of 11S upon heating treatment. (**A**) Size volume distributions of 11S and 11S-MEL-A. (**B**) FTIR spectrogram of 11S and 11S-MEL-A with heat treatment (0.25 h and 6.0 h). The native protein served as a control.

The size volume distributions of 11S and 11S-MEL-A solutions were analyzed to illustrate the effects of MEL-A on the structure of 11S. As shown in Figure 3A and Figure S1A, the average diameter of 11S is less than 100 nm, which was consistent with an early report [31]. 11S gathers into some irregular shape when heating at 95 °C for 0.25 h. The longest side of the 11S aggregates exceeds 1 μm (Figure S1B from Supplementary Materials). This was also confirmed by the size volume distribution results (Figure 3). As the heating time increased, the structure of the aggregates was still irregular (Figure S1D from Supplementary Materials). In addition, the larger aggregates with a size above 1 μm accounted for the major proportion with the heating time increasing to 1.0 h and 3.0 h, which the average size above 1.5 μm increased. The heated water solution of globular proteins can form aggregates with four distinct morphologies: spheres-shaped granules, flexible chain structure, amyloid (semi-flexible) fiber structure, and irregular aggregation structure (amorphous). The aggregates formed during heating treatment have two distinct forms: spherical particles with diameters between 50 nm and a few microns or chains with diameters less than 10 nm and lengths between tens of nanometers and tens of microns. These changes have been well described in the literature, which suggests that spherical aggregates were induced at pH 7.0 and 95 °C [32]. In general, under neutral pH conditions, the hydrophobic groups inside the heat-treated protein molecules are partially exposed. When the electrostatic repulsion is the greatest and the net charge is quite high, the chains are generated. At the same time, spherical particles are produced when approaching the isoelectric point of the protein. Upon higher protein concentrations, chains and spherical particles tend to reassemble, finally resulting in larger shapes, and the conformation is randomly assembled.

In the 11S solution doped with MEL-A, spherical particles were formed after 0.25 h, 1.0 h, and 3.0 h heat treatment (Figure 3A). The size-volume distribution shows the unimodal distribution. At a longer heating time, the scattering of 11S and 11S-MEL-A has an obvious difference. The results showed that MEL-A reduces the size of 11S aggregates, which are usually formed under heat treatment conditions, indicating that the thermal stability of 11S is enhanced. Generally, the aggregation of 11S/soy protein proceeds by using physical interactions, which may involve hydrogen bonding and hydrophobic interactions. The use of MEL-A might induce bindings between hydrophobic acetyl or fatty acid chains with nonpolar groups of protein side chains, therefore, leading to protein denaturation to promote the new self-assembling spheroidal structures. It was inferred that the micelle structure of the surfactant acted as an alternative interface, which prevented proteins from self-aggregation [33]. As presented in Figure S1E from Supplementary Materials, the aggregates embedded into a vesicle structure were observed to be similar to the “core-shell” structure. Owing to its molecular structure, MEL-A, with a concentration above its CMC, is prone to self-assemble into large vesicles in solutions [34], and 11S aggregates can easily insert into the vesicles through molecular interactions [13,15]. In this report, the interactions between MEL-A and 11S aggregates are prone to occur, leading to core-shell particle formation. Moreover, upon heat treatment, increasingly more hydrophobic groups buried inside the native protein became exposed.

### 2.4. FTIR Characterization

To elucidate the biosurfactant-protein interaction occurring between 11S and MEL-A, Fourier transform infrared spectrometer (FTIR) was used to determine the interaction. As data shown in Figure 3B, the typical characteristic absorption bands of 11S changed considerably upon heat treatment. With the aggregation proceeding, the broad intense absorption peak at 1652 cm^−1^ presented 80% C=O, and 20% C-N was increased. The peaks at 1540 cm^−1^ assigned to the 60% N-H, 30% C-N, and 10% C-C stretching vibration were greatly obvious as well. The broad intense absorption band ranging from 1000 to 950 cm^−1^ decreased as well. The use of MEL-A makes all the absorption peaks change, especially at the range from 1000 to 500 cm^−1^ and the range at 2850–2960 cm^−1^ region were due to the symmetric and asymmetric stretching modes of methylene (CH_2_) and methyl (CH_3_) groups found in aliphatic chains of proteins [35]. These results show that MEL-A changes the secondary structure of 11S.

### 2.5. Circular Dichroism Spectroscopy Determination

**Figure 4:**
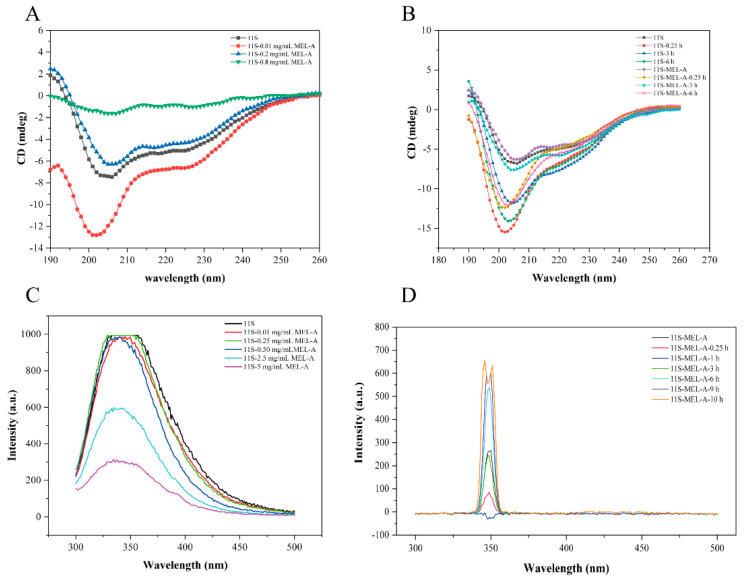
Effect of MEL-A on the secondary structure of 11S protein upon heating treatment. (**A**) CD spectra of 11S in the absence and presence of MEL-A at different concentrations ranging from 0 to 5.0 mg/mL. (**B**) CD spectra of 11S and 11S-MEL-A after heating treatment ranged from 0 to 6.0 h. The changes in the secondary structures are summarized in charts. (**C**) Effects of different MEL-A concentrations (0, 0.01, 0.25, 0.5, 2.5, and 5.0 mg/mL) on the fluorescence intensity of 11S. The inset presents the Stern–Volmer plots describing the fluorescence quenching of 11S in the presence of MEL-A. (**D**) The fluorescence intensity changes of 11S with the addition of 0.5 mg/mL MEL-A (above CMC) after heat treatment.

Usually, Circular dichroism (CD) is adopted to analyze the secondary structural changes of the protein. We used CD to further illustrate the secondary structure changes of 11S caused by MEL-A treatment and heating. As shown in Figure 4A and Table 1, the untreated 11S had a distinctly negative peak at 205 nm. The addition of 0.01 mg/mL MEL-A made the negative peak lower than the addition of 0.2 and 0.8 mg/mL MEL-A. The positive peak at 190 nm showed opposite results without the addition of MEL-A. It was inferred from Figure 4B that the band intensity of 11S was largely increased through a short heating treatment for 0.25 h, while the influence was weaker when the heating time was 3 h and stronger at the heating time of 6 h (Table 2). Moreover, the CD trend in 11S-MEL-A mixtures was the same. Furthermore, the calculations were carried out in the software. Concerning control 11S, the increased helix was formed according to the duration of heat treatment. Since the helix-rich regions of the 11S sequence had a greater propensity to form aggregates, MEL-A may disperse the 11S aggregates by affecting the helix of the 11S protein.

### 2.6. Fluorescence Quenching of 11S Induced by MEL-A

Figure 4C shows the effects of 0–5 mg/mL MEL-A on the intrinsic fluorescence intensity of 11S. The fluorescence emission band of 11S was about 350 nm. The shift was inconspicuous when the concentration of MEL-A was 0.01 mg/mL, 0.25 mg/mL, 0.5 mg/mL. However, with an increasing concentration of MEL-A, a quenching of fluorescence progressively appeared. This fluorescence attenuation should be concerned with the MEL-A-induced conformational changes of 11S. Generally, there are several interpretations for fluorescence quenching, such as molecular rearrangement, energy transfer, and dynamic or static quenching. The fluorescence intensities of 11S-MEL-A mixtures concerning heating time were analyzed in Figure 4D. Compared with the control 11S in Figure 4C (fluorescence intensity at 350 nm was about 1000 a.u.), the strength of the 11S-MEL-A mixtures appeared to be weaker after heat treatment. The intensity of the 11S-MEL-A mixtures decreased sharply at the beginning of the heat treatment (the heating time increased from 0 h to 1 h), while the peak value of the mixture did not change (Figure 4D). When the heating time reached 3 h, the fluorescence intensity of 11S-MEL-A was comparable to that of the unheated mixture. However, the fluorescence intensity of the mixture increased as the heating time continued to increase (6–10 h), at which time the fluorescence emission peak of 11S-MEL-A was divided into two parts. At this point, the exact reason for this is not clear and remains to be further explored.

### 2.7. Quantitation Measurement of MEL-A and 11S Interaction by ITC

**Figure 5:**
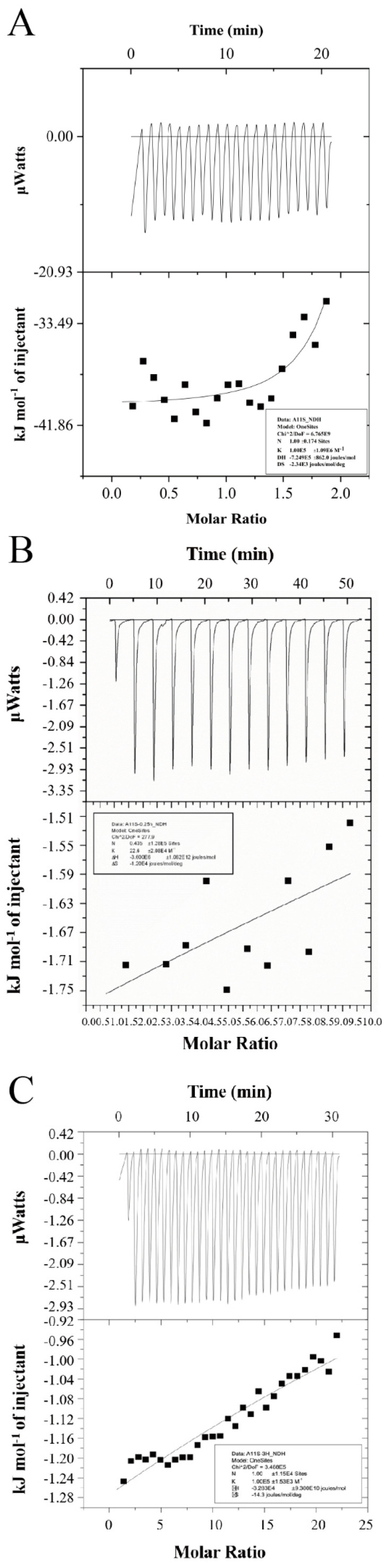
ITC determination of the binding of MEL-A to 11S. The upper panel shows heat flows obtained upon injection of MEL-A solution into the 11S solution at 25 °C. The lower panel indicates the molar heat values obtained through the integration of the individual heat flow signals as a function of the MEL-A concentration. (**A**) native 11S; (**B**) 11S heating 0.25 h; (**C**) 11S heating 3.0 h.

We used Isothermal titration calorimetry (ITC) to quantitatively measure the interaction between MEL-A and 11S. As can be seen from the data in Figure 5A, the enthalpy of exothermic release during titration tends to plateau. It was concluded that MEL-A micelle can combine with 11S to form micellar clusters on the surface of 11S to form exothermic peaks. This was due to the saturation of the bindings. The fitting results also confirmed this characteristic. The ITC data of the One Sites model were fitted with thermodynamic parameters. The binding ratio (N) is 1.00 ± 0.174, indicating that 11S is in equilibrium and about one MEL-A binding molecule per mole. According to the results of the transmission electron microscope (TEM) and CD, the structure of 11S aggregates had changed. The interaction may be due to the concentration of 10 mM MEL-A in the calorimeter syringe being much higher than CMC. That makes MEL-A combine with 11S in the form of an aggregation phase (micelle and bubble) to form a non-aggregation and core-shell structure. This was consistent with other surfactants, such as the 11S binding of stevioside [36], suggesting that MEL-A micelle formation reduces protein aggregation. The calculated affinity constant K = 1.00 × 10^5^ ± 1.09 × 10^6^ M^−1^ was higher than the high-affinity constant K_a_ > 10^4^ M^−1^; thus, the interaction between the two molecules was binding. ΔH = −7.249 × 10^5^ ± 862.0 J/mol and ΔS = −2.34 × 10^3^ J/mol/K also prove strong interaction behavior. As mentioned earlier, electrostatic or hydrophobic interactions were the primary driving force for the binding of surfactants to proteins. In the present case, the combination of MEL-A and 11S was more likely to be driven by hydrogen bond formation. This was consistent with the exothermic binding process detected by ITC. Moreover, the interaction between MEL-A and the hydrophobic domains in the protein was nonspecific, which was consistent with the findings of fluorescence, FTIR, and CD examinations.

We also analyzed the binding between MEL-A and the heat-denatured proteins, as shown in Figure 5B,C. The heat trace peaks similarly tended to plateau. We used the site model to express. The increasing number of MEL-A molecules bound per mole of 11S is N = 1.35 ± 1.48 (Figure 5B) and N = 1.00 ± 1.15 × 10^5^ (Figure 5C). This high binding ratio further supported the formation of MEL-A clusters or micelles at the surface of 11S. Interactions with binding constant K_a_ > 10^4^ M^−1^ were considered to be of high affinity. As a result, the binding constant observed in this study indicated that the binding of MEL-A with 11S was probably suggesting the forming of a hydrogen bond.

## 3. Discussion

In this work, the effect of a glycolipid biosurfactant, mannosylerythritol lipid-A (MEL-A), on the heat-induced soy glycinin (11S) aggregates were investigated. The interfacial behaviors, as well as the foaming and emulsifying properties of heat-induced 11S, were also determined. The results showed that MEL-A at concentrations higher than the CMC reduced the random aggregation of 11S protein after heat treatment, facilitating the formation of self-assembling core-shell particles composed of a core of 11S aggregates covered by a shell of MEL-A vesicles. After heat treatment, a significant decrease in surface tension at the air-water interface, viscoelasticity, and foaming ability was observed, while MEL-A improved the foaming stability and emulsifying properties of 11S aggregates. Infrared spectroscopy, circular dichroism spectroscopy, fluorescence spectroscopy, and isothermal titration calorimetry were used to prove the interaction forces between MEL-A and 11S protein, which were driven by hydrophobic interactions between the exposed hydrophobic groups of the protein and fatty acid chains or acetyl groups of MEL-A, as well as the hydrogen bonding between mannosyl-D-erythritol groups of MEL-A and amino acids of 11S. This study focuses on the interactions between MEL-A and 11S, revealing its multiple potential applications as a natural food additive and emulsion in the beverage and bakery industries.

## 4. Materials and Methods

### 4.1. Microorganisms and Chemicals

MEL-A was produced from *Moesziomyces aphidis* DM34 fermentation. Soy flour was purchased from Shandong Yuwang Industrial and Commercial cooperation in China. The protein content of soy flour was 55.10% (determined by the micro-Kjeldahl method, N × 6.25, dry basis). 11S globulin was prepared as the description by Yuan et al. [37]. The protein content of 11S globulin was 96.4%. All other chemicals used were of analytical grade.

### 4.2. Structure Identification

The surface morphology of the 11S and 11S-MEL-A mixtures was examined by JEM 1200 transmission electron microscope at 60 kV (JEOL, Tokyo, Japan). Nano Zs90 Zetasizer instrument (Malvern Instruments, Worcestershire, UK) was adopted to measure the particle size of 11S, and the 11S-MEL-A mixture was determined. All measurements were conducted at least three at 25 °C.

### 4.3. Interaction between MEL-A and 11S

#### 4.3.1. Infrared Spectroscopy

We used a Fourier transform infrared spectrometer (Nicolet, Rhinelander, WI, USA) to analyze the characteristic groups of 11S heated aggregates and MEL-A. The transmittance was recorded from 4000 to 500 cm^−1^.

#### 4.3.2. Circular Dichroism Spectroscopy

The sample of 0.2% 11S was made with the 0.01 mM pH 7.0 phosphate buffer solution. The structural changes of 11S with MEL-A at different concentrations (0–1.0 mg/mL) and 11S by the addition of 0.5 mg/mL MEL-A after heat treatment of 0–9.0 h. CD spectroscopy was performed at 25 °C using an instrument J-1500–150ST (JASCO, Tokyo, Japan) from 195 nm to 300 nm. Each measurement was repeated three times. The structural parameters were analyzed by Dichroweb.

#### 4.3.3. Isothermal Titration Calorimetry

ITC measurements were carried out using a VP-ITC titration calorimeter from MicroCal (Malvern Instruments, UK) to obtain the interaction of MEL-A toward 11S. The mixing cell was filled with a 0.05 mM native, heating 0.25 h and heating 3.0 h 11S solution. We filled the calorimeter syringe with a MEL-A (10.0 mM) solution. The program parameters were 19.0 μL total injection, cell temperature 25 °C, stirring speed 307 rpm, filter period 2 s, and the reference power 10 μcal/s. Dripping the MEL-A into PBS was controlled. All data were processed with the Origin in the VP-ITC titration calorimeter. The modified data were fitted into a graph of heat flux (μcal/s) and time (min) and then normalized to obtain the enthalpy curve (ΔH, kcal/mol) per mole of MEL-A. Finally, we obtained the thermodynamic parameters of K (binding constant), ΔH (enthalpy change), ΔS (entropy change), and N (number of MEL-A molecules bound per mole of 11S) based on the fitted one-site independent binding model using the TA Nano Analyzer software in the equipment.

#### 4.3.4. Fluorescence Spectroscopy

A fluorescence spectrometer (Cary Eclipse, Varian, Santa Clara, CA, USA) was used to study the fluorophore of 11S-MEL-A. 0.1% 11S solutions were mixed with 0, 0.01, 0.5, 1.0, 2.5, 5.0, and 10 mg/mL MEL-A in 0.01 mM, pH 7.0 phosphate buffer. The parameters of the fluorescence spectrometer are as follows: emission wavelength is 290–460 nm, the excitation wavelength is 295 nm, and slit width is 5 nm. Then used the Stern–Volmer equation to calculate the quenching rate constant and the quenching process. Moreover, we studied the effects of MEL-A on the fluorophore of the heated 11S. 0.2% 11S heated for 0–10 h mix with or without 0.5 mg/mL MEL-A. The parameters of the fluorescence spectrometer are the same as before.

### 4.4. Functional Properties

#### 4.4.1. Dynamic Surface Tension

The dynamic surface tension of heated 11S and 11S-MEL-A mixtures at the air-liquid interface was examined using an Automatic Surface Tensiometer (DropMeter A-100P, Haishu Maishi Testing Technology Co., Ltd., Ningbo, China). All measurements were carried out at 25 °C. The surface tension (γ) was recorded in 1800 s. The surface pressure (π) was expressed as the difference between the surface tension of PBS (0.1 mM, pH 7.0, γ0) and γ. The surface dilatational modulus (E) was chosen to display the interfacial rheological behavior of the mixtures. Its changes were dependent on the surface pressure and time.

#### 4.4.2. Foaming Properties and Emulsifying Properties

FC and FS are two important indicators to evaluate protein foam. FC is defined as the ability of a protein to bind air to a protein solution, which can be determined by the increase in the volume of foam. FS is the ability to maintain the desired appearance of the foam, which can be measured by the rate of foam volume reduction [38]. FC and FS of 11S or 11S-MEL-A after heat treatment were determined as described by Gravel et al. with some modifications [39]. A volume of 10 mL 11S (2%, *w*/*v*) solution with or without 0.5 mg/mL MEL-A was placed in a 50 mL measuring cylinder. Then, the solutions were agitated at a speed of 15000 rpm for 1 min at ambient temperature using a high-speed Ultraturrax T25 homogenizer (IKA, Labortechnik, Staufen, Germany) to produce a foam. After blending, the total volume of foam was measured immediately at time 0 (V_0_). The calculation formula of FC and FS are presented as follows:

FC (%) = (V_0_ − 10)/10 × 100 (1)

FS (%) =(V_t_ − 10)/(V_0_ − 10) × 100 (2)

V_0_ is the total volume of foam directly after blending, and V_t_ is the volume after standing for t min.

The EAI and ESI of 11S solution (0.2%, *w*/*v*) after heat treatment were measured. 10 mL soy oil and 30 mL protein solution with or without 0.5 mg/mL MEL-A homogenized using a high-speed homogenizer (IKA, Labortechnik, Germany) with 15,000 rpm for 1 min. Drew 50 μL fresh emulsion from the bottom (0.5 cm) of the beaker using a pipettor and then mixed with 5.0 mL of 0.1% (*w*/*v*) sodium dodecyl sulfate solution. The absorbance of the diluted emulsion was recorded at 500 nm. Then the absorbance of the diluted emulsion was measured again after standing for 10 min. EAI and ESI were calculated as follows:

EAI (m^2^/g) = 2 × 2.303 × A_0_ × N/C/Ø/10,000 (3)

ESI (min) = A_0_/(A_0_ − A_10_) × 10 (4)

N is the dilution factor, N = 100, C is the weight of the protein per unit volume (g/mL), and Ø is the oil volume fraction of the emulsions. A_0_ and A_10_ are the absorbance of the emulsion at 0 and 10 min.

### 4.5. Statistical Analysis

The results were expressed as the average values from three parallel experiments. The one-way analysis of variance (ANOVA) and the Duncan tests (SPSS 20) was used for data analysis. *p* < 0.05 was regarded as statistically significant.

## 5. Conclusions

In conclusion, we investigated the interfacial behavior and emulsification properties of 11S-MEL-A mixtures. Higher concentrations of MEL-A combined with 11S exhibited a synergistic effect on the interfacial tension, which decreased when MEL-A was present. These effects could be attributed to the interaction between 11S and MEL-A. The long-term stability of emulsions allowed resistance in response to external deformations. Understanding the long-term properties of stabilized emulsions aids its application in the food industry. Future study of protein-LMW surfactant interactions is imperative to understand the chemical-biological properties and interfacial performance of 11S-MEL-A hybrid emulsions, which have multiple potential applications as natural food additives and emulsions in various beverage and bakery industries.

## Figures and Tables

**Table 1:** Prediction values of protein secondary structure upon different heating times.

Samples	Heating Time/h	Helix/%	Strand/%	Turns/%	Unordered/%	Total/%
11S	0	28.6	5.1	15	51.3	100
	0.25	40.7	0	3.9	55.5	100.1
	3	43.4	0.8	7.2	48.6	100
	6	45.8	0	5	49.2	100
11S-MEL-A	0	40.1	1.8	8.2	49.8	99.9
	0.25	32	0	4.9	63.1	100
	3	26.3	0.3	5.4	68	100
	6	39.9	0	6	54.1	100

**Table 2:** Prediction values of protein secondary structure upon different MEL-A concentrations.

Samples	Concentration of MEL-A/mg/mL	Helix/%	Strand/%	Turns/%	Unordered/%	Total/%
11S	0	28.6	5.1	15	51.3	100
	0.01	40.1	0	0.4	59.5	100
	0.2	40.1	1.8	8.2	49.8	99.9
	0.8	14.5	21.9	14.6	49	100.1

## Data Availability

The data presented in this study are available in the article and Supplementary Material.

## Supplementary Materials

The following supporting information can be downloaded at: https://www.mdpi.com/article/10.3390/molecules27217393/s1, Figure S1: TEM micrographs of 11S and 11S-MEL-A. (A) 1.0% 11S. (B) 1.0% 11S after heat treatment for 0.25 h. (C) 1.0% 11S solutions added with 0.5 mg/mL MEL-A after heat treatment for 0.25 h. (D) 1.0% 11S after heat treatment for 3 h. (E) 1.0% 11S solutions added with 0.5 mg/mL MEL-A after heat treatment for 3 h.
