# Supplementary material for: Interactions between Mannosylerythritol Lipid-A and Heat-Induced Soy Glycinin Aggregates: Physical and Chemical Characteristics, Functional Properties, and Structural Effects
Source: Molecules. 2022 Oct 31;27(21):7393. doi: 10.3390/molecules27217393 (PMC9657017; doi:10.3390/molecules27217393)

molecules

Supplementary Materials

Interactions between Mannosylerythritol Lipid-A and Heat-Induced Soy Glycinin Aggregates: Physical and Chemical Characteristics, Functional Properties, and Structural Effects

Siyu Liu , Hongyun Lu, Xiayu Liu, Ying Shi * and Qihe Chen *

Department of Food Science and Nutrition, Zhejiang University, Hangzhou 310058, China
* Correspondence: shiying0520@zju.edu.cn (Y.S.); chenqh@zju.edu.cn (Q.C.); Tel.: +86-139-6717-1522 (Q.C.)

Figure S1. TEM micrographs of 11S and 11S-MEL-A. (**A**) 1.0% 11S. (**B**) 1.0% 11S after heat treatment for 0.25 h. (**C**) 1.0% 11S solutions added with 0.5 mg/mL MEL-A after heat treatment for 0.25 h. (**D**) 1.0% 11S after heat treatment for 3 h. (**E**) 1.0% 11S solutions added with 0.5 mg/mL MEL-A after heat treatment for 3 h.

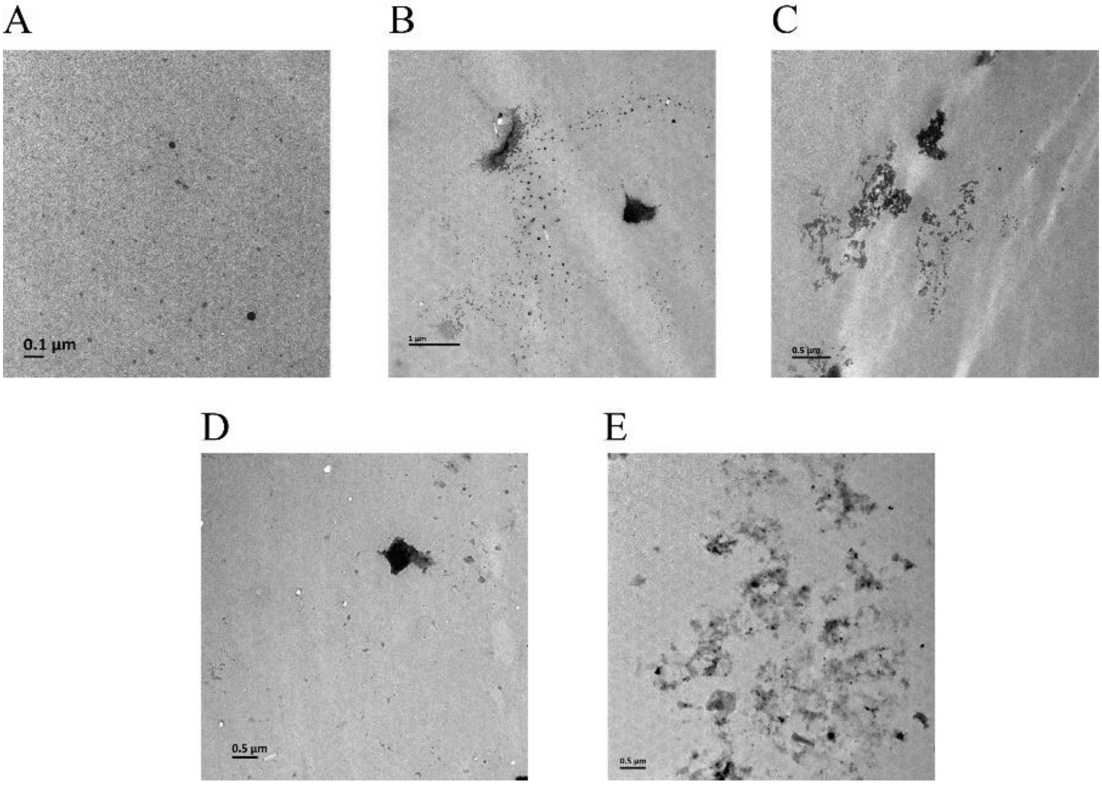

Supplement: Supplementary file 1 [file molecules-27-07393-s001.zip › molecules-1975689-supplementary.pdf]
